# Neural block therapy for radiation enteritis: a case report

**DOI:** 10.1186/s40981-019-0239-9

**Published:** 2019-03-13

**Authors:** Moegi Tanaka, Yoshinori Kamiya, Hiroki Shimizu, Tatsunori Watanabe, Natsuko Naito, Hiroshi Baba

**Affiliations:** 1 Division of Anesthesiology, Niigata University Graduate School of Medical and Dental Sciences, 1-757 Asahimachi-dori, Chuo ward, Niigata City, 951-8510 Japan; 2 Department of Anesthesiology, Niigata University Medical and Dental Hospital, 1-757 Asahimachi-dori, Chuo ward, Niigata City, 951-8510 Japan

**Keywords:** Radiation enteritis, Ileus, Inferior mesenteric artery plexus block, Splanchnic nerve block

## Abstract

**Background:**

Radiation enteritis following radiotherapy targeting the abdomen occasionally causes ulcers or ileus, which can be difficult to treat and usually progressive and refractory, significantly degrading the patient’s quality of life.

**Case presentation:**

A 58-year-old woman had undergone surgery for cervical cancer approximately 21 years ago. During treatment, she had also received radiotherapy targeting the pelvis and stomach. She presented with complaints of vomiting and lower abdominal pain and was subsequently diagnosed with multiple gastric ulcers, enterocolitis, and paralytic ileus due to late radiation-induced sequelae. We reasoned that visceral sympathetic block would improve the abdominal symptoms; therefore, we performed a splanchnic nerve block and an inferior mesenteric artery plexus block. As predicted, these block procedures improved the symptoms.

**Conclusions:**

Radiation enteritis is an iatrogenic disease, and there is no established treatment for intractable cases. However, visceral sympathetic nerve block may show efficacy as a potential therapy for radiation enteritis-associated abdominal pain and ileus.

## Background

Radiation enteritis following radiotherapy targeting the abdomen occasionally causes ulcers or ileus, which can be difficult to treat. We report on the case of a woman suffering from paralytic ileus and abdominal pain due to radiation enteritis as a result of radiotherapy after previous cervical cancer surgery. Splanchnic nerve block and inferior mesenteric arterial plexus block procedures improved both the abdominal pain and the ileus symptoms.

## Case presentation

We obtained comprehensive consent beforehand from the patient, and we obtained consent to report the clinical experience and publish the accompanying images.

The patient was a 58-year-old woman suffering from lower abdominal pain and vomiting, which gradually worsened from the start of December 2017. An upper gastrointestinal endoscopy revealed multiple gastric ulcers and scars, and lower gastrointestinal endoscopy revealed inflammation in the large intestine. The patient had undergone surgery in 1997 for cervical carcinoma, followed by postoperative radiotherapy that involved irradiation of her pelvis and stomach. On the basis of the symptoms, endoscopy findings, and medical history, the patient was diagnosed with refractory multiple gastric ulcers, enterocolitis, and paralytic ileus due to late radiation-induced sequelae.

As a result of frequent vomiting, the patient had become unable to ingest orally. She was hospitalized in October 2018 and fasted before being placed under central venous nutrition control. Hyperbaric oxygen therapy was started to treat the intractable multiple gastric ulcers and ileus. Thirty treatment sessions were scheduled over a period of approximately 1–2 months. Pentazocine and tramadol were administered to treat the abdominal pain, but these achieved poor results.

**Fig. 1:**
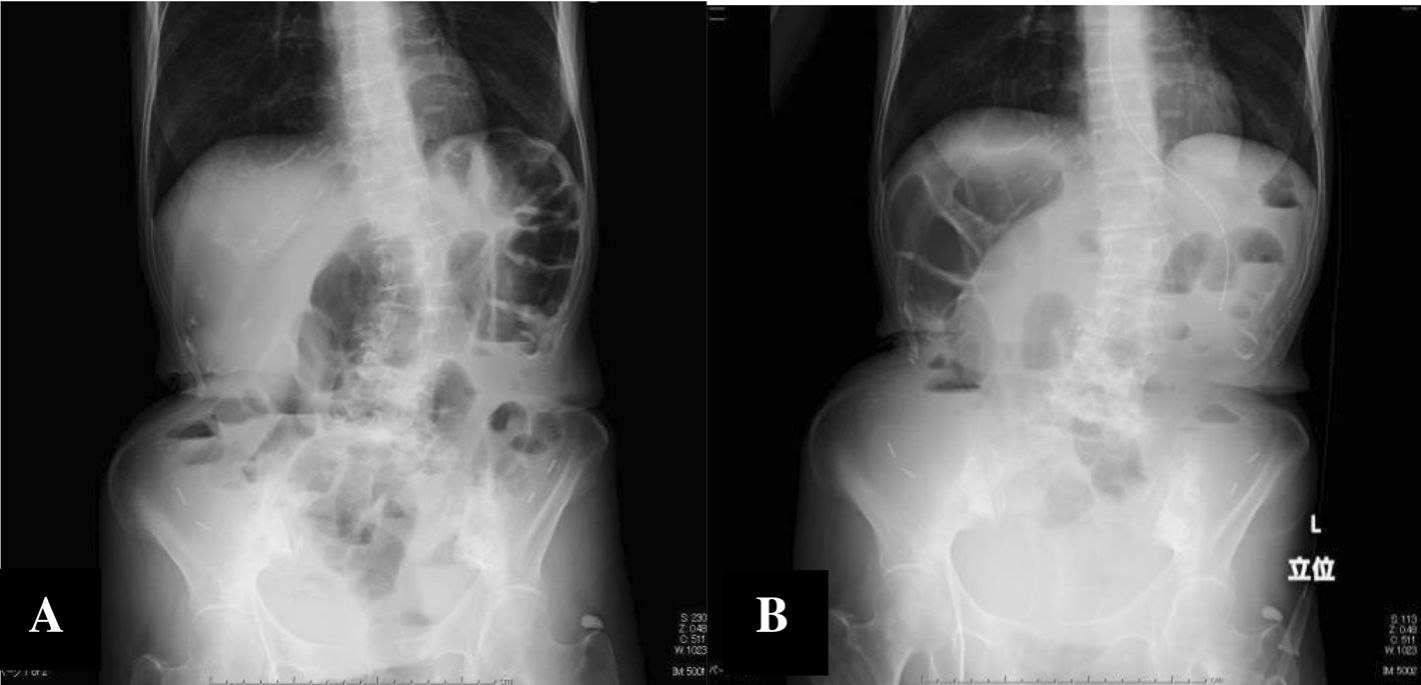
**a** Patient’s abdominal X-ray at hospitalization. It shows several small intestinal niveau and extension of the colon and small intestine by intestinal gas. **b** Patient’s abdominal X-ray after the inferior mesenteric artery plexus block. The number of intestinal niveau and the amount of gas has decreased, as compared to before the block

At hospitalization, the patient was 155 cm tall and weighed 43 kg. Although she exhibited abdominal distention, no muscular defense or rebound pain was observed. On percussion, the bowel sound was attenuated. A blood examination revealed anemia, slight liver dysfunction, low protein levels, and hypoalbuminemia. An abdominal X-ray showed several small intestinal niveau and extension of the colon and small intestine by intestinal gas (Fig. 1a).

The patient experienced increased lower abdominal pain during bowel movements and felt relieved after defecation. Consequently, we hypothesized that her abdominal pain was due to poor peristalsis of the intestinal tract and that blocking the sympathetic nerve of the intestinal tract would help normalize the peristaltic movement and relieve the pain. The pain was strongest in the lower abdomen at the level of the 12th thoracic nerve. An epidural catheter was inserted via the lumbar1–2 intervertebral space, and continuous epidural anesthesia with 0.2% ropivacaine at 4 ml/h was administered for 1 week, resulting in good control of the lower abdominal pain and defecation. This allowed the tramadol, which promotes constipation, to be discontinued. A block of the inferior mesenteric artery plexus with ethanol was performed, resulting in an immediate improvement in the patient’s bowel movements and defecation, and a reduction in lower abdominal pain, now measured as 3 on a numerical rating scale (NRS = 3). An abdominal X-ray was performed after the block procedure, showing the number of intestinal niveau and the amount of gas had decreased, as compared to that before the block (Fig. 1b). The control of lower abdominal pain was effective, and defecation was noted. Tube feeding was started 4 days after the inferior mesenteric artery plexus block, and oral feeding was started 2 days later.

**Fig. 2:**
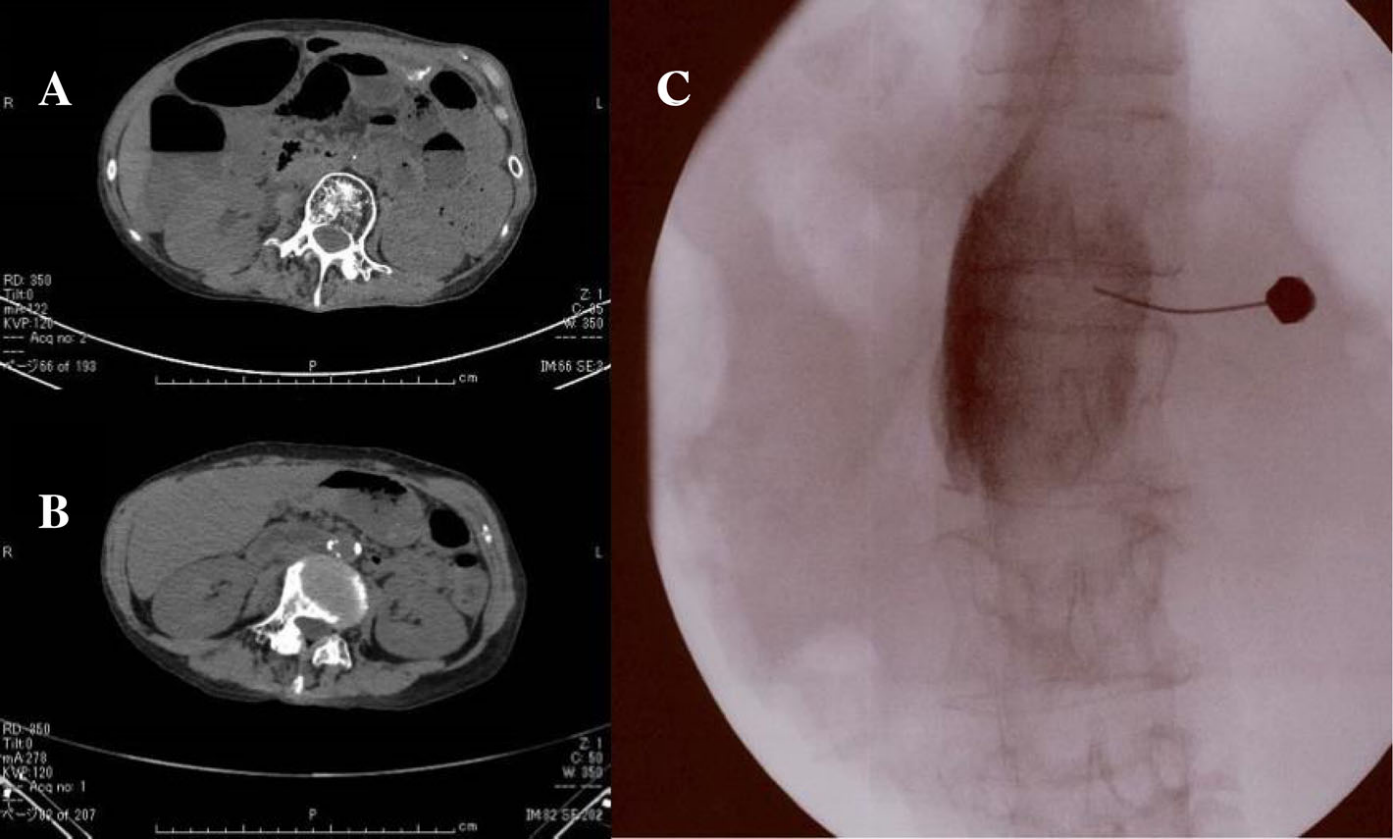
**a** Patient’s abdominal CT. It shows dilation of the small intestine, as well as the formation of niveau. **b** Patient’s abdominal CT after the splanchnic nerve block. It shows a large improvement in the ileus after the block. **c** A splanchnic nerve block with ethanol was performed

Twelve days after the block, the patient’s lower abdominal pain increased to NRS 8 and there was recrudescence of the vomiting. An abdominal computed tomography (CT) scan showed dilation of the small intestine, as well as the formation of niveau; however, no bowel obstruction or disruption of blood flow was observed (Fig. 2a).

An upper gastrointestinal contrast examination with contrast medium (Gastrografin®, Bayer Yakuhin Ltd., Osaka, Japan) showed relatively good peristaltic movement of the large and small intestine but poor gastric peristaltic movement. A large amount of food residue was also observed. An epidural catheter was inserted via the thoracic 6–7 intervertebral space for innervation of the upper gastrointestinal tract, including the stomach and small intestine, and continuous epidural anesthesia with 0.2% ropivacaine at 4 ml/h was started. This alleviated the abdominal pain, and the vomiting disappeared.

**Fig. 3:**
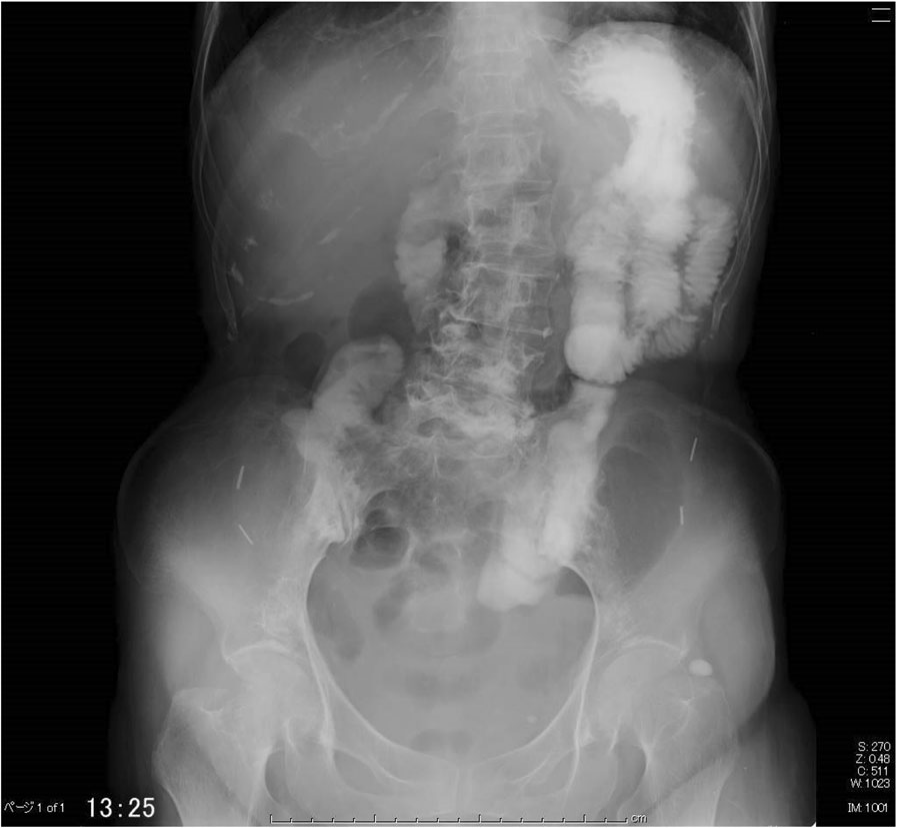
An upper gastrointestinal contrast examination shows that the peristaltic movement of the upper gastrointestinal tract and the stomach had improved after the splanchnic nerve block

Approximately 2 weeks later, continuous epidural anesthesia was stopped, and a splanchnic nerve block with ethanol was performed (Fig. 2c). This resulted in an improvement in the lower abdominal pain to NRS 1, as well as an increase in food intake. The following day, CT showed a large improvement in the ileus (Fig. 2b). An upper gastrointestinal contrast examination showed that peristaltic movement of the upper gastrointestinal tract had improved (Fig. 3). The subsequent course was good, and the hyperbaric oxygen therapy was completed. The patient was discharged from hospital at the end of January. At the outpatient visit 2 weeks later, the general condition of the patient was good and she could orally intake her normal diet.

## Discussion

Radiation enteritis is an iatrogenic disease, with no established treatment for intractable cases. However, the results of the present case suggest that a sympathetic nerve block procedure in the abdomen, such as a splanchnic nerve block or inferior mesenteric plexus block, may improve symptoms associated with radiation enteritis. It is widely accepted that these blocks are effective for intractable abdominal pain, but there has been no previous report about their effects on ileus. To the best of our knowledge, this is the first report detailing the therapeutic benefit of block procedures in the treatment of ileus by enhancement of the peristaltic motion of the intestines through suppression of the sympathetic nervous system.

Radiation enteritis caused by radiation therapy is classified as either early or late according to whether it occurred within 3 months of or 6 months after irradiation, respectively. Early radiation enteritis manifests as reversible injury of the intestinal mucosa and watery diarrhea, with normal recovery after the irradiation ends. On the other hand, late radiation enteritis is thought to be caused by intestinal ischemia and fibrosis caused by radiation endarteritis and subsequent thrombotic microcirculatory disturbances, causing pathological conditions such as enteritis and ileus. Late radiation enteritis is clinically problematic; the late sequelae are usually progressive and refractory, significantly degrading the patient’s quality of life [1].

The radiation enteritis in the present case developed more than 10 years after radiotherapy treatment, similar to a previous case report that describes the sudden development of radiation enteritis after a long period of time [2]. In our case, the radiation therapy was performed in the pelvis after cervical carcinoma surgery and gastric lymphadenopathy. We concluded that the enteritis, ileus, and refractory multiple gastric ulcers were most likely to be late sequelae of the irradiation because of her history of radiotherapy, consistency of the irradiation site, and the ulcer site. In addition, it has previously been reported that the risk of paralytic ileus increases after pelvic surgery [1, 2].

When the late sequelae include stenosis, perforation, and fistula formation in the intestinal tract, surgical treatment is recommended. However, the present case was not considered for surgical treatment because there was no evidence of occlusion or stenosis of the intestinal tract. Moreover, surgery should be avoided where possible because it promotes the adhesion of the intestinal tract. In this case, there was no routine treatment option; the patient was administered hyperbaric oxygen therapy, inhaling oxygen at a high concentration to raise the environmental pressure to 2 atm or more using hyperbaric oxygen therapy apparatus. Hyperbaric oxygen therapy is an established treatment for conditions such as gas gangrene, carbon monoxide poisoning, cerebral infarction, and intestinal obstruction [3–7]. It has also been reported to be effective in ameliorating the symptoms of ileus arising as a consequence of late sequelae following radiotherapy [3, 4, 8–11]. However, hyperbaric oxygen therapy can be time-consuming. In the present case, the patient underwent 30 treatment sessions, with little change in her subjective symptoms. For patients with a diminished quality of life, receiving only hyperbaric oxygen therapy over a long time period can be mentally stressful.

In this case, abdominal sympathetic systems were implicated in the patient’s ileus. Previous studies have shown that sympathetic nerve blocks increase intestinal blood flow and improve intestinal motility and wall tension [12, 13]. Moreover, the multiple gastric ulcers revealed during the initial examination of the patient were observed to be almost healed in the final upper gastrointestinal endoscopic examination before discharge. This finding indicates that concurrent treatment with hyperbaric oxygen therapy and splanchnic nerve block, which suppresses the sympathetic nerve controlling the stomach and the blood flow, contributes to healing refractory gastric ulcers.

In conclusion, in this case of paralytic ileus caused by radiation enteritis, a splanchnic nerve block and a mesenteric arterial block were performed for analgesic purposes and to release the ileus. Sympathetic nerve blocks for abdominal organs may offer one of the few treatments for radiation enteritis.

## Abbreviations

CT: Computed tomography
NRS: Numerical rating scale
